# Retro walking treadmill training reduces C—reactive protein levels in overweight and obese young adults: A randomized comparative study

**DOI:** 10.1002/hsr2.1169

**Published:** 2023-03-30

**Authors:** Ajith Soman, Sunil Chandy, Khalid Alkhathami, Baranitharan Ramamoorthy, Bijad Alqahtani

**Affiliations:** ^1^ Department of Health Rehabilitation, College of Applied Medical Sciences Shaqra University Shaqra Saudi Arabia; ^2^ Department of Clinical Lab Science, College of Applied Medical Sciences Shaqra University Shaqra Saudi Arabia

**Keywords:** arterial blood pressure, biochemical marker, life style induced illness, treadmill, walking

## Abstract

**Background and Aims:**

Retro walking has been shown to acquire a greater metabolic cost, placing a higher cardiopulmonary demand on the body, when compared with forward walking at a similar speed. The aim of this study was to compare the effect of retro walking with that of forward walking on C‐reactive protein (CRP) levels, body mass index (BMI) and blood pressure (BP) and to understand the influence of independent factors namely systolic blood pressure (SBP), diastolic blood pressure (DBP) and BMI on CRP in untrained overweight and obese young adults.

**Methods:**

This was a randomised controlled trial whereby 106 participants underwent either retro walking (*n* = 53) or forward walking (*n* = 53) treadmill training four times a week for 12 weeks before and after which CRP, BMI, and BP levels were measured. Comparison of the measured values before and after intervention and between the groups was done and influence of BMI and BP on CRP levels was determined.

**Results:**

Both groups recorded a significant decrease (*p* < 0.001) in CRP, BMI, and BP levels postintervention. The participants who underwent retro walking training showed a significantly (*p* < 0.001) higher decrease in all the outcomes as compared with the forward walking group. C‐reactive protein levels were seen to be influenced by BMI and DBP.

**Conclusion:**

Retro‐walking training leads to greater decrease in CRP, BMI, and BP when compared with forward walking, and CRP levels are influenced by BMI and DBP. Retro walking treadmill training can be used preferentially to bring about reduction in cardiovascular risk factors.

## 1 INTRODUCTION

Obesity is an epidemic escalating across the globe. Obese individuals suffer from a higher fatality rate of 50%–10% from multiple causes, especially cardiovascular events.^1^ Additionally, they suffer from a low‐grade chronic proinflammatory state, which is the factor that links endothelial dysfunction to insulin resistance and obesity. C‐reactive protein (CRP), an important inflammatory marker in serum, has been reported to be elevated in persons who are obese, and correlates with insulin resistance and endothelial dysfunction.^2^ Obesity or an increase of fatty tissue causes chronic inflammation in the body, which in turn causes an increase in cytokine synthesis.^3^ The level of CRP in blood at an acute stage is an early marker of inflammation or infection, and has been associated with central obesity. CRP is normally produced by the liver in response to IL‐6 (Interleukin‐6) in acute inflammatory conditions, and the normal concentration of this acute‐stage protein in the blood is less than 0.3 mg/dl.^4^, ^5^, ^6^ A small amount of CRP is also generated by cells other than those in the liver; atherosclerotic plaques, lymphocytes, monocytes, and neurons, for instance.^7^ Furthermore, CRP is most consistently associated with atherogenesis, especially by macrophages and smooth muscle cells, when compared with other inflammatory markers, and hence poses a greater cardiovascular risk.^7^, ^8^ Many studies have demonstrated the interrelationship of CRP, BMI, and blood pressure (BP), though this relationship has not been conclusively proven.^9^, ^10^

It is a widely accepted fact that obesity is not much amenable to medical treatment. Diet and exercise, systematically undertaken, is the only way to treat obesity, manifested as a high body mass index (BMI). Of the different modes of exercise, walking at a brisk pace is beneficial to the cardiovascular health and helps in maintaining a healthy body weight.^11^, ^12^ Retro walking or backward walking performed on a treadmill has been proven to expend energy at a greater extent than forward walking. Retro walking also makes a higher metabolic demand on the body and helps in improving exercise capacity to a greater extent than forward walking.

The discrepancy in metabolic cost among the two types of walking is postulated due to increased stride frequency, decreased stride length and also owing to the concentric contraction of the quadriceps muscle as opposed to eccentric contraction, resulting in increased energy expenditure after retro walking.^13^, ^14^ These effects may indirectly have an effect on CRP and BMI, since inflammatory and obesity markers are linked to exercise capacity and energy expenditure.

The feasibility of retro walking as a possible mode of exercise in robust adults has been analyzed in several studies. Terblanche et al., when studying the metabolic energy expenditure of backward running and walking, established that running and walking backward caused higher submaximal heart rates, blood lactate levels, and oxygen consumption responses, when compared with running forward at a similar velocity.^15^ Likewise, Ordway inferred that retro walking can not only produce performance gains, but also favorably influence parameters like disease‐related disability and pain in persons with specific disease conditions.^16^

The authors of the present study identified a need to compare the effectiveness of backward walking as opposed to forward walking in bringing about changes in inflammatory outcome measures, especially CRP, which is an important inflammatory marker with great influence on atherogenesis and hence on development of lifestyle diseases. Both forms of walking are exercise modes that are easy to administer, and are potentially able to modify risk factors for cardiovascular disease. CRP is likely to be influenced by a number of factors like BMI, SBP and DBP, and it is important to identify the independent variables which can influence CRP. Keeping this aim in mind, the present study was conducted with the primary objective of ascertaining which forward walking and retro walking treadmill training had greater effect on CRP level in the blood among obese and untrained young men. The secondary objective was to determine the independent variables which were liable to influence blood CRP levels.

## 2 MATERIALS AND METHODS

### 2.1 Subjects

The study included male subjects aged between 20 and 25 years, whose BMI was equal to or above 25 kg/m^2^. We excluded persons who were undergoing drug therapy for any medical conditions, smokers, those currently participating in any habitual exercise training, those who had any metabolic, orthopedic, neurological or respiratory conditions, which contraindicated exercise training or any apparent medical condition that causes hepatic interference of CRP secretion.

The demographic data of the study participants, which included age, clinical data including height, weight, BMI, history of past illness, and physical activity profile was recorded. A complete physical examination was carried out for the participants. Those not fulfilling inclusion criteria were withdrawn from the study.

### 2.2 Study design

This study was a single‐blinded comparative pre–post experimental study without control. Young male students pursuing medical and paramedical courses from Shaqra University, Kingdom of Saudi Arabia, who volunteered to participate in the study upon being solicited by advertisements placed on the University's notice boards, e‐mails, and posts in the social media students’ groups were included as participants. Approval to conduct the study was obtained from the ethics research committee of the university (ERC_SU_20210057). Reporting of results was done in accordance with the CONSORT guideline recommendations for reporting clinical trials.

### 2.3 Recruitment

Before initial assessment, participants received a data package with details of the study, including their rights as participants of a research project, and a printed informed consent form. In addition, the participants were explained about the procedure, and were encouraged to clarify any doubts about the same before signing the written consent form. Once the form was signed, the subjects were allotted into two groups using sequentially numbered sealed opaque envelopes and assessment and interventions were carried out. Before intervention, the participants were screened using the pre‐participation questionnaire recommended by the American Heart Association/American College of Sports Medicine.^17^ BMI was calculated, BP was measured, and CRP levels were ascertained by laboratory testing.

### 2.4 Intervention

Before initializing the intervention, basic training was provided by a physical therapist to those who were unfamiliar with forward and backward training, until they could walk confidently on the treadmill without support. Participants in either group underwent 5 min of warm‐up and cool–down each before and after intervention which comprised free range of motion exercises of all joints, heel raise exercises, hamstring stretching, and soleus stretching.

The participants allotted to the retro walking group participated in a backward treadmill training program under supervision for 4 days a week for 12 weeks. Each session included an exercise period, which started with duration of 15 min, and progressed to 30 min over the 12‐week training period. During the exercise period, the participants were made to walk backwards at a speed of 4 km/h (or 67 meters/min) with a 10% gradient^18^, ^19^ The participants in the forward walking training group underwent a supervised treadmill training program with duration, intensity, and frequency similar to that of the retro walking treadmill training program.

### 2.5 Assessment and outcomes

Outcomes of the exercise program viz. BMI, CRP, and BP levels were measured by assessors who were trained by the research team and blinded to the objectives of the study, to the intervention groups to which the participants belonged, and to the phase of measurement, that is, preintervention or postintervention. All measures were evaluated the day before commencement of the training program and the day after the end of the training period.

Blood samples were obtained from the antecubital vein after an overnight 12‐ hour fast and immediately before commencement of the treadmill exercise. The post‐exercise blood samples were taken 24–72 h following the last exercise session.

The fluorescence immunochromatographic method (Wiz biotech®) was used to measure the CRP levels from the blood samples.^20^ Body height, body weight, BMI, hip circumference, waist circumference, waist–height ratio and waist–hip ratio were measured using standardised methods.^21^, ^22^

The BP was measured from the right arm using the auscultatory method, with a sphygmomanometer with a cuff size appropriate for arm length. Four readings were taken with the participant seated, the arm supported on a cushion at chest level, resting a minute between each measurement. The mean of the last three readings was considered the final level of BP.^9^

### 2.6 Sample size

To expect an improvement in the CRP level of 1.94 ± 0.9 after exercise, on the basis of the study conducted by Arikawa et al.,^22^ with 95% connfidence interval (CI) and 90% power with an allowable error of 5%, a minimum of 49 individuals per group were to be recruited.

## 3 STATISTICAL ANALYSIS

The data were analysed using statistical software SPSS version 21 (SPSS, Inc.). Baseline data are represented as mean and standard deviation with 95% CI. Group (Retro walking vs. Forward walking) × Time (Baseline vs. 12 Week); mixed model analysis of variance (ANOVA) was used to assess the effect and interactions between the groups. Assumptions for normality, homogeneity of variance, and sphericity of the covariance matrix, when assessed, showed no major deviations. Independent *t* test was used to analyse the between group characteristics at baseline and a *p* < 0.05. was assumed to be significant.

The postintervention data were clubbed together and multiple linear regression analyses were performed to find the influence of independent variables (BMI, DBP, and SBP) on the dependent variable (CRP). Assumptions for normality, multicollinearity, and homoscedasticity, when assessed, showed no major deviations. The strength of relationship between the dependent and independent variables was assessed using the beta (*β*) standardized coefficient. To investigate the mediation effect of DBP and SBP on CRP a simple mediation analysis was performed using PROCESS.

## 4 RESULTS

**Figure 1:**
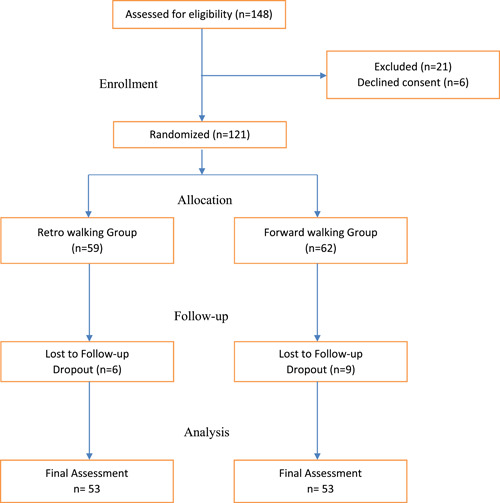
CONSORT flowchart.

A total of 148 participants were assessed for eligibility, of which 21 did not fulfill inclusion criteria and were excluded and 6 participants refused to participate during the initial screening. Randomization into groups was carried out for 121 participants, of which 15 dropped out during the course of the intervention. The final data was recorded from 106 participants of which 53 were from the forward walking group and 53 from the retro walking group. Figure 1 presents a CONSORT flow chart.

The mean age of participants was 21.32 ± 1.73 with a mean BMI of 32.70 ± 4.88. The baseline characteristics of the subjects are presented in Table 1.

**Table 1:** Baseline characteristics of the groups.

Variable	Forward Walking (*n* = 53)	Retro Walking (*n* = 53)	*p* (95% CI)
Age	21.32 ± 1.73	21.47 ± 1.54	0.63 (−0.47 to 0.78)
BMI	32.70 ± 4.88	32.12 ± 4.37	0.52 (−2.37 to 1.21)
Waist height ratio	0.58 ± 0.07	0.56 ± 0.06	0.06 (−0.05 to 0.0001)
Waist hip ratio	0.86 ± 0.07	0.83 ± 0.06	0.09 (−0.05 to 0.0003)
DBP	84.19 ± 3.26	83.11 ± 2.48	0.06 (−0.38 to 2.19)
SBP	129.37 ± 13.92	130.90 ± 4.82	0.44 (−2.45 to 5.58)
CRP	4.29 ± 3.33	3.46 ± 2.48	0.15 (−1.96 to 0.30)

Abbreviations: BMI, body mass index; CI, confidence interval; CRP, C reactive protein; DBP, diastolic blood pressure; SBP, systolic blood pressure.

### 4.1 Group and time interactions

Both groups showed significant improvements in all the outcomes during the 12‐week intervention. The mixed model ANOVA results for (Group × Time) interaction was significant for BMI (*p* < 0.001, F 48.75), DBP (*p* < 0.001, F 26.25) SBP (*p* < 0.001, F 13.52), and CRP (*p* < 0.001, F 26.34) (Table 2).

**Table 2:** Between‐group analysis of outcomes in forward walking and retro walking.

	Pretreatment		Posttreatment		*p*	
Outcome	Forward walking	Retro walking	Forward walking	Retro walking	Time	Time × group
BMI	32.70 ± 4.88	32.12 ± 4.37	29.76 ± 4.70	27.23 ± 3.52	<0.0001	<0.0001
DBP	84.19 ± 3.26	83.11 ± 2.48	82.45 ± 3.12	78.98 ± 1.55	<0.0001	<0.0001
SBP	129.37 ± 13.92	130.90 ± 4.82	128.71 ± 5.32	123.40 ± 3.90	<0.0001	<0.0001
CRP	4.29 ± 3.33	3.47 ± 2.48	3.67 ± 2.96	1.47 ± 1.02	<0.0001	<0.0001

Abbreviations: BMI, body mass index; CRP, C reactive protein; DBP, diastolic blood pressure; SBP, systolic blood pressure.

### 4.2 Multivariate relationships

Table 3 illustrates the findings of multiple regression analysis: BMI (*β*: 0.432, *p* < 0.0001, 95% CI: 0.156–0.337) and DBP (*β*: 0.317, *p* < 0.0001, 95% CI: 0.078–0.441) were the significant predictors of CRP.

**Table 3:** Multiple linear regression analysis for the factors influencing CRP.

	Unstandardized coefficient		Standardized coefficient		95% CI		Model summary	
	B	SE	*β*	*p* Value	Lower bound	Upper bound	*R* ^2^	*p* Value
Constant	−31.899	4.712		<0.0001	−41.245	−22.554	0.538	<0.0001
BMI	0.246	0.047	0.432	<0.0001	0.156	0.337		
DBP	0.260	0.092	0.317	0.005	0.078	0.441		
SBP	0.051	0.053	0.112	0.338	−0.054	0.157		

Abbreviations: BMI, body mass index; CI, confidence interval; CRP, C reactive protein; DBP, diastolic blood pressure; SBP, systolic blood pressure.

### 4.3 Mediation effect

**Figure 2:**
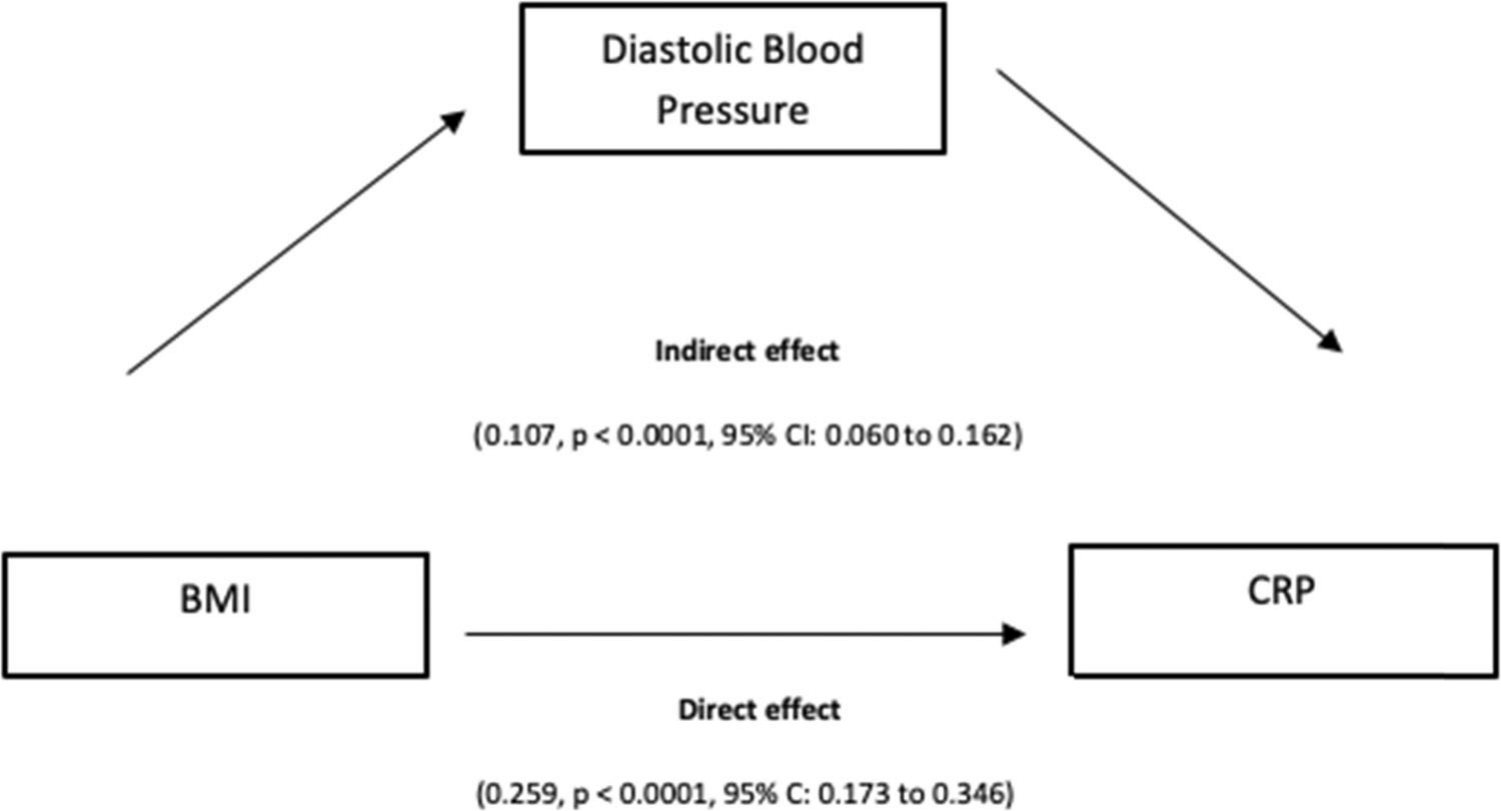
Mediating role of diastolic blood pressure in the relationship between body mass index and C reactive protein.

The results of mediation effect of DBP on BMI and CRP have been descripted in Figure 1. The total effect of BMI on CRP was (0.366, *p* < 0.0001, 95% CI: 0.281– 0.451) with a direct effect of 0.259, *p* < 0.0001, 95% CI: 0.173–0.346 and an indirect effect of 0.107, *p* < 0.0001, 95% CI; 0.060–0.162 (Figure 2).

## 5 DISCUSSION

The primary aim of our study was to compare the effects of retro walking and forward walking training on blood CRP level and BMI of untrained young men. The results showed that both parameters decreased significantly in both groups following treatment. In addition, systolic and diastolic BP were analyzed, which were both seen to be reduced after normal walking and retro walking. The effect of retro walking in reducing the level of CRP over the period of intervention, BMI, DBP, and SBP was greater than that of forward walking. The variables SBP, DBP, and BMI were seen to influence CRP levels.

At a similar level of intensity, backward walking places higher demands on metabolic sensorimotor, cardiovascular, and perceptual responses than forward walking does. Also, backward walking poses a considerable challenge to standing dynamic balance, thus recruiting more neurons in the process.^18^, ^23^ Hyun‐Gyu and co‐workers found that backward walking stimulated the lower limb muscles and resulted in higher energy consumption in the lower limbs. They also stated that backward walking stimulates the quadriceps muscles and other muscles which subsequently move the knee joint in a considerably more balanced manner as compared with forward walking.^24^ Owing to the increased challenge to the different systems of the body, retro walking increases energy expenditure relative to forward walking. This increased energy expenditure causes a decrease in the level of adiposity in the body, thus leading to decreased body weight and consequently decreased BMI, as proven in the results of the present study. Similarly, several researchers have found that retro walking produces more energy expenditure than forward walking at similar speeds.^18^, ^23^, ^25^ Arikawa et al. exclaimed that a 16‐week aerobic exercise regime in obese females significantly reduced levels of CRP.^22^

The CRP is an inflammatory marker and it is well‐documented that obese individuals express a chronic inflammatory status and that accounts for elevated CRP levels in this category. Persons who suffer from obesity or hyperinsulinemia tend to produce a higher amount of CRP from adipocytes along with other inflammatory markers.^26^ CRP levels have been seen to be higher in overweight and obese persons when compared with those who were not.^27^, ^28^ In the present study as well as in some previous studies BMI also has been identified to be a strong predictor of elevated CRP levels.^29^, ^30^

Exercise and physical activity has been known to reduce levels of CRP by increasing levels of adiponectin‐ a relatively novel anti‐inflammatory adipocytokine known to improve insulin sensitivity. Leptin is yet another polypeptide, which is closely associated with CRP levels and is decreased with physical activity and exercise. Exercise in general, can decrease levels of adipose tissue and leptin levels and increase adiponectin levels, ultimately leading to decreased CRP levels.^26^, ^31^

Physical exercise has been seen to have an influence on the immune system in that it reduces the number of mononuclear cells in blood, which in turn produce proinflammatory cytokines like (IL1, IL‐6, IL‐8, and CRP).^32^ Moderate exercise done regularly can decrease CRP and IL‐6 levels in obese persons.^33^ Exercise has an anti‐inflammatory effect which can reduce systemic inflammation and CRP levels. In agreement to this, the present study also saw a decrease in CRP levels following both modes of walking.

As an exercise mode, which places more demand on the cardiovascular and metabolic system than regular walking, it could be expected that retro walking would have a similar effect, but of more magnitude, on CRP levels. The present study is the first in our knowledge to evaluate the effect of a backward walking program on an inflammatory marker and cardiovascular risk factor such as the CRP in young obese and preobese individuals. Terblanche et al. have found that a backward walking program can increase levels of cardiovascular fitness and produce changes in body composition.^34^ Similarly, a meta‐analysis demonstrated that physical training can be correlated to reduced CRP levels regardless of age or gender, and that greater improvements in CRP levels could be seen additionally when the BMI is reduced.^35^ In contrast, Mouridsen et al. noted that there was a spike in high‐sensitivity CRP as an immediate response to exercise, however, the increase was moderate and not independently associated with coronary artery disease.^36^

**Figure 3:**
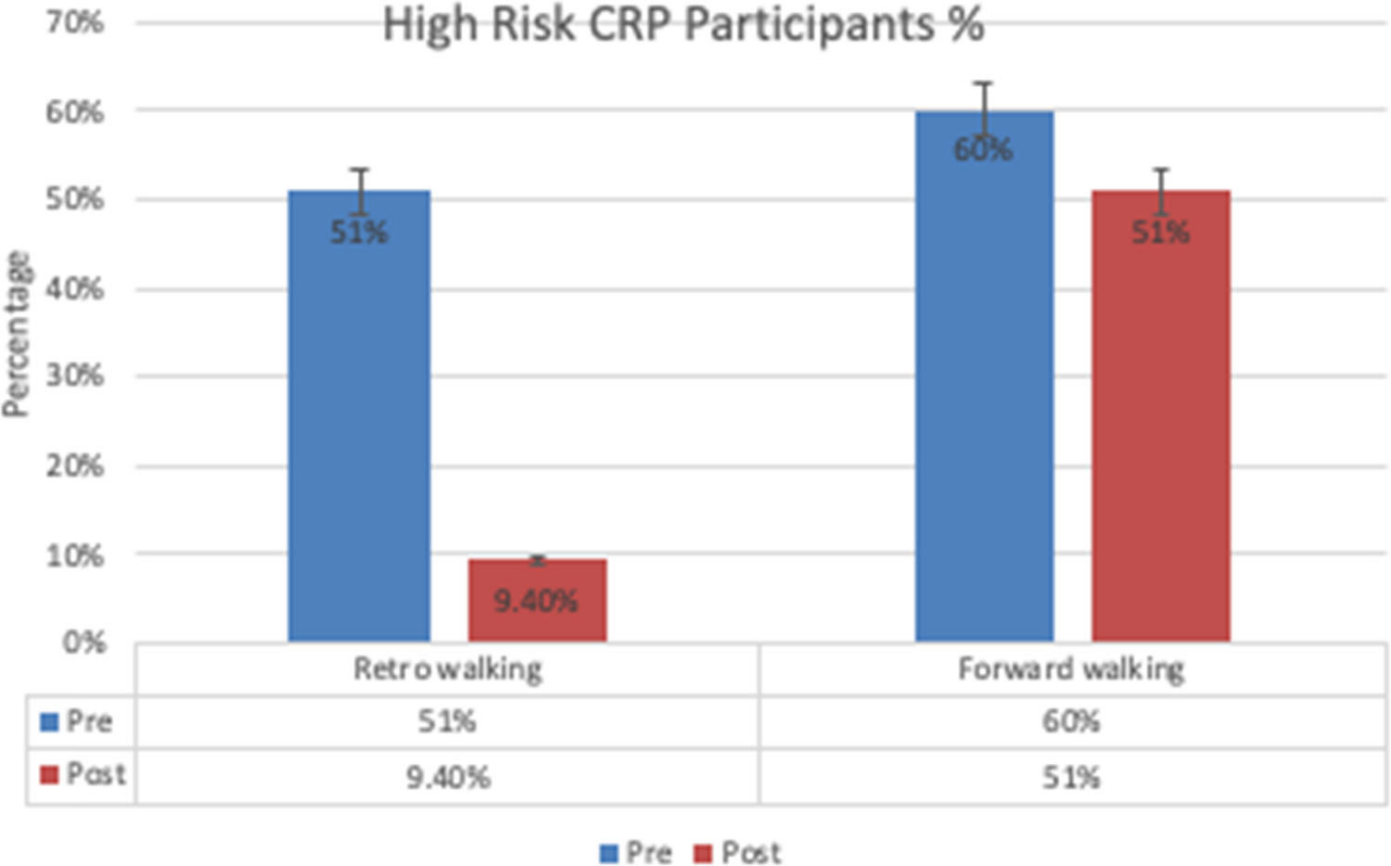
Pre‐ and post‐exercise high risk (CRP > 3 mg/L) CRP values. BMI, body mass index; CRP, C reactive protein.

The multiple linear regression analysis revealed that BMI and DBP were the significant predictors of CRP. Studies have demonstrated CRP to be associated with obesity, increased waist circumference and systolic BP; these parameters can be used for identification and intervention in children and adolescents with high risk of atherosclerosis.^9^ An expanding body of evidence indicates that inflammation has a major role to play in the development of high BP; elevated levels of CRP have been shown to be associated with the incidence of hypertension in middle‐aged adults.^37^, ^38^ In the present study, most of those who had stage 1 hypertension were in the high‐risk CRP category (CRP > 3 mg/L), and conversely, none of those with high‐risk CRP were with normal BP. (Figure 3) This would demonstrate the importance of CRP as a predictor of cardiovascular risk factors, including hypertension and ischemic heart disease.^39^ The study of factors, which can modify CRP levels in the body would play an important role in prevention and management of cardiovascular risk factors.

Walking is an activity, which is said to be a complex interaction, which envelopes sensory, physiological and mechanical input to produce results that are optimum.^40^ An important advantage of retro walking over forward walking is that it places less stress on the joints of the lower limb, which is an important factor to be considered during exercise in obese persons. Also, more energy can be expended in a shorter period of time, which makes the exercise more time efficient. Walking backwards was a novel task for most of the participants, and during the initial stages of the training, many expressed apprehension to walk backwards, especially on the treadmill.

In the between‐groups comparison, there was a significant difference in the outcomes between the forward‐walking and retro‐walking groups. The present study adds to the existing body of literature to provide evidence about the benefits of retro walking by documenting a reduction in CRP, which is not only an important inflammatory marker, but also a cardiovascular risk factor. Retro walking also places less stress on the weight‐bearing joints of the lower limb, and thus reduces the probability of injury during exercise in people who are overweight or obese. It can be surmised that retro walking is a viable replacement to forward walking in reducing cardiovascular risk factors.

The strengths of the present study were the relatively high sample size and the documentation of CRP levels, which is a less used outcome measure in study of effect of retro walking, as an outcome measure for the treatment program in obese and pre‐obese young adults. The limitations include lack of a control group, which would monitor any fluctuations in CRP levels in the absence of activity, lack of monitoring the diet of participants, which could have had a major effect on CRP levels and lack of an objective measure such as oxygen consumption or VO_2_ for measuring the intensity of exercise. Also, the noninclusion of women in the study sample might result in lack of generalizability of the study results across genders. This study did not address the long‐term effects of the exercise, hence we could not prove if the exercise would have a lasting effect on the CRP level after the intervention period of 12 weeks.

## 6 CONCLUSIONS

The present study demonstrates that both retro walking and forward walking can help alleviate CRP level and obesity. However, the retro walking program has added benefits over a forward walking program of similar intensity in modifying these outcomes. BMI and DBP have the potential to influence CRP levels in the blood. These factors can be considered while designing an exercise program to modify cardiovascular risk factors in young individuals. Considering the advantages and practicability of use, retro walking can be a valuable addition to any exercise program which aims at addressing obesity and cardiovascular risk factors.

## AUTHOR CONTRIBUTIONS

**Ajith Soman**: Conceptualization; data curation; formal analysis; funding acquisition; investigation; methodology; project administration; resources; software; supervision; validation; writing—original draft; writing—review and editing. **Sunil Chandy**: Conceptualization; formal analysis; funding acquisition; investigation; methodology; project administration; resources; validation; writing—review and editing. **Khalid Alkhathami**: Conceptualization; funding acquisition; investigation; methodology; resources; validation; writing—original draft; writing—review and editing. **Baranitharan Ramamoorthy**: Data curation; software; supervision; writing—review and editing. **Bijad Alqahtani**: Data curation; funding acquisition; methodology; resources; writing—original draft; writing—review and editing.

## CONFLICT OF INTEREST STATEMENT

The authors declare no conflict of interest.

## ETHICAL STATEMENT

The study was conducted according to the guidelines of the Declaration of Helsinki, approval to conduct the study was obtained from the ethics research committee of the university (ERC_SU_20210057).

## TRANSPARENCY STATEMENT

The lead author Ajith Soman affirms that this manuscript is an honest, accurate, and transparent account of the study being reported; that no important aspects of the study have been omitted; and that any discrepancies from the study as planned (and, if relevant, registered) have been explained.

## Data Availability

The data that support the findings of this study are available from the corresponding author upon reasonable request.
